# Aorto-Renal Dysplasia in Childhood: The Overlap of Neurofibromatosis Type 1 and Pediatric Renovascular Hypertension

**DOI:** 10.1007/s11906-025-01350-7

**Published:** 2026-03-16

**Authors:** Eden Singh, Kevin Meyers, Dawn M. Coleman, Santhi K. Ganesh

**Affiliations:** 1 Section of Vascular Surgery, Department of Surgery, Duke University, Durham, USA; 2 Division of Nephrology, Department of Pediatrics, Children’s Hospital of Philadelphia and University of Pennsylvania, Philadelphia, USA; 3 Division of Cardiovascular Medicine, Department of Internal Medicine, University of Michigan Medical School, Ann Arbor, USA; 4 Department of Human Genetics, University of Michigan Medical School, Ann Arbor, USA; 5 Department of Internal Medicine, University of Michigan, MSRB III, 1150 West Medical Center Drive, Ann Arbor, MI 7220, 48109-0644 USA; 6 Duke University School of Medicine, Durham, USA

**Keywords:** Pediatric, Renovascular hypertension, Neurofibromatosis, Aorto-Renal dysplasia

## Abstract

**Purpose of Review:**

This narrative review aims to summarize what is currently understood about Neurofibromatosis Type 1 (NF-1) and renovascular hypertension (RVH) in children, including clinical presentation and diagnosis, epidemiology, genetics, and management considerations including advances in treatment modalities.

**Recent Findings:**

Most of what is currently understood about NF-1 and arterial dysplasia leading to RVH relies on the inclusion of patients with NF-1 in single-institution reports. The management of pediatric RVH often requires multi-modal therapies inclusive of anti-hypertensive medications and revascularization for refractory cases, through catheter-based (i.e., endovascular) and open surgical means. There is a need to develop genotype-targeted guidelines for the diagnosis and management of pediatric aorto-renal dysplasia resulting in RVH in patients with NF-1.

**Summary:**

While our understanding of pediatric RVH and NF-1 has evolved over the past decade, critical research questions have emerged that encompass epidemiology, etiology and genetics. These research questions require immediate attention to establish and optimize standardized diagnostic and treatment guidelines.

## Introduction

Pediatric renovascular hypertension (pRVH) is believed to account for 8–10% of hypertension in children [1]. Pediatric non-atherosclerotic arterial fibrodysplasia as a result of developmental etiologies is the most common cause of aorto-renal occlusive disease [2]– [3]. Isolated suprarenal aortic narrowings, including supravalvar aortic stenosis (SVAS) and mid-aortic syndrome (MAS), may co-occur in children with renal artery disease, and all forms may alter kidney blood flow, reducing perfusion that is responsible for renin-mediated blood pressure elevation, often refractory to drug therapy [1, 3–5]. If not diagnosed and appropriately treated, pRVH may challenge development and risk critical end-organ damage [6]. While pediatric arterial dysplasia most commonly affects the renal arteries and abdominal aorta, it may also involve mesenteric, cervical (i.e., carotid or vertebral), upper and lower extremity, and intra-cranial vasculature [7].

The rates of genetic syndromes among patients with pRVH vary in the literature. Syndrome diagnoses associated with pRVH range in frequency but are all considered relatively rare and include diseases such as Neurofibromatosis Type 1 (NF-1), Williams Syndrome, Alagille Syndrome, Tuberous Sclerosis, and others. NF-1 is caused by pathogenic variation in the *NF1* gene and has an estimated prevalence 1:2052 [8]. Patients with NF-1 and pRVH are well-described in the literature as they have been observed with greater frequency in clinical series of pRVH compared to other syndromes [9–11]. For example, in a University of Michigan surgical series (*N* = 169) patients with NF-1 comprised 18% of the cohort, and in a Boston Children’s surgical series (*N* = 39) comprised 15.4% of patients with MAS [9]– [12]. Conversely, the frequency of pRVH diagnosis among those with NF-1 appears to be low. In a series examining the incidence of peripheral vascular lesions in patients with NF-1 (*N* = 2,322), a 0.69% incidence of peripheral vascular lesions was reported [13]. In patients with NF-1 that do have aorto-renal dysplasia, it has been found that the renal arteries are affected in 41% of patients [14]. Genetic studies of pRVH and/or MAS to date have reported a subset of patients with defined genetic syndrome diagnoses (14–43%) [15, 16] and corresponding expected pathogenic gene variants, including *NF1* gene variants in individuals with NF-1 clinical diagnoses [15].

The diagnosis and treatment of pRVH is not yet well-informed by clinical trials nor formal clinical practice guidelines, in part due to its rarity. Institutional practice patterns vary, although there are some widely accepted best practices. Those considerations in rare genetic syndromes are often influenced by specific arterial pathophysiology, as well as other considerations of co-morbid diagnoses. This review of current literature aims to summarize the current presentation, genetic basis, and treatment outcomes in patients with NF-1 and pRVH.

## Disease Classification

pRVH is a manifestation of an array of vascular occlusive disease presentations that result in diminished renal perfusion, thus stimulating renin-mediated angiotensin release. Diminished renal perfusion causes the juxtaglomerular cells of the kidney to release renin and subsequently over-activate the renin-angiotensin-aldosterone system [17]. Pediatric aorto-renal dysplasia as a result of developmental etiologies is the most common cause of renal artery stenosis in children and related RVH in North America and Western Europe [1, 4, 18, 19]. Inflammatory vasculitis is another common cause of RVH, with Takayasu arteritis being the most common cause of RVH in Asia and South Africa [4, 18]. It has been proposed that developmental pRVH is caused by pathologic processes that are distinct from those that underlie multifocal fibromuscular dysplasia (FMD), a disease also characterized by non-atherosclerotic arterial fibrodysplasia, with recurrent luminal narrowings and mural dilations, leading to the classic “string-of-beads” seen on angiographic imaging [19].

Significant differences have been described between pediatric unifocal disease and multifocal FMD in terms of histopathology, clinical symptoms, anatomic phenotype (i.e., imaging findings), and response to treatment [4, 7, 11, 19, 20]. An analysis of the 33 pediatric patients in the United States Registry for fibromuscular dysplasia demonstrated that the renal arterial vasculature was involved in 97% of the children included in the FMD registry [7]. It also was found that the mean age of diagnosis of FMD in children was 8.4 years, and the cohort of 33 patients was 62% female [7]. While the terminology “pediatric FMD” has been used in the literature, we propose that unifocal FMD of the renal arteries in children would be better referred to as a manifestation of a spectrum of aorto-renal dysplasia that presents in children, particularly in pre-teens. Unifocal FMD in children has several distinct differences from multifocal FMD that may manifest in older children. Additionally, the presence of concurrent MAS or SVAS in children raises the need for nomenclature that more broadly encompasses the aorta (Table 1).

**Table 1:** Arterial dysplasia subtypes. NF-1, neurofibromatosis type 1; RVH, renovascular hypertension; LVH, left ventricular hypertrophy; CHF, congestive heart failure; FTT, failure to thrive

Arterial Dysplasia Subtype	Dominant Angiographic Findings	Histologic Findings	Age Group	Molecular Associations (gene names)	Common Clinical Presentation
NF-1 with Midaortic Syndrome, Renal Artery Stenosis, or Both	Usual focal ostial renal artery stenosis, abdominal aortic narrowing	Intimal fibroplasia, disruption of the internal elastic lamina, and thinning of the media	Infants-Children	*NF1*	Systemic hypertension/RVH most frequently described, concurrent arterial pathology includes aneurysms and Moyamoya, LVH with CHF may be observed, FTT
Isolated Midaortic Syndrome	Usual suprarenal or intrarenal abdominal aortic narrowing	Intimal fibroplasia with or without internal elastic fragmentation and duplication, medial elastic disorganization or focal medial fibrosis; rarely, luminal thrombus	Infants-Children	*NF1*, *JAG1*, *NOTCH2*, *ELN*, *TSC1*, *TSC2*	Systemic hypertension/RVH most frequently described, LVH with CHF may be observed, FTT
Isolated Renal Artery Stenosis	Focal renal artery stenosis (ostial, mid, or distal/branch)	Intimal and fibroplasia, and/or adventitial fibroplasia infrequently	Infants-Children	*NF1*, *JAG1*, *NOTCH2*, *ELN*, *TSC1*, *TSC2*, *YY1AP1*, *PDGFRB*, *NOTCH3*	Systemic hypertension/RVH most frequently described, LVH with CHF may be observed, FTT
Multifocal Fibromuscular Dysplasia	Multifocal narrowings and intervening mural dilations in an artery	Medial fibroplasia	Teens*-Adults	Complex genetic architecture (*COL5A1*, *PTIGR* in rare cases)	Systemic hypertension/RVH, stroke, mesenteric ischemia, myocardial infarction, LVH with CHF may be observed

* anecdotal report of very rare children under age 13

Among patients with NF-1, the spectrum of arterial dysplasia includes MAS and renal artery stenosis (RAS). RAS in NF-1 can involve occlusive disease at any location from the renal artery ostium to second-order branches, but most commonly affects the ostial and proximal segments [14]. Concurrent MAS and RAS have been reported more frequently in NF-1 compared to other forms of pRVH [14].The arteriopathy in NF-1 reflects proliferative arterial remodeling, a non-neoplastic manifestation of NF-1. Associated malignant transformation has not been described. NF-1 is often diagnosed clinically based on characteristic findings such as café au lait spots, axillary and inguinal ephelides, and cutaneous neurofibromas [21]. Other manifestations of NF-1 include plexiform neurofibromas, optic gliomas, pheochromocytoma, paraganglioma, pulmonic valve stenosis, and urinary tract anomalies [10, 21–24]. The diagnosis of aorto-renal dysplasia and pRVH is often delayed and made upon finding elevated blood pressure during screening that is recommended annually in NF-1; thus, pRVH is typically diagnosed after a diagnosis of NF-1 has been established [25]– [26].

There is a pressing need for better disease classification in NF-1, particularly through molecular and genetic approaches, to support studies of the natural history of NF-1-associated arterial dysplasia and pRVH.

### Genetics and Molecular Mechanisms of NF-1-Related Aorto-Renal Dysplasia

NF-1 is an autosomal dominant disorder caused by germline mutations in the *NF1* gene, which encodes neurofibromin [21]. Neurofibromin functions as tumor suppressor by downregulating the RAS signaling pathway [27]. It is expressed in both vascular endothelial and smooth muscle cells, implicating it in the development of arteriopathy in patients with NF-1 [21]. NF-1-related arteriopathy has been linked to reduced *NF1* mRNA expression, activation of the Mitogen Activated Protein Kinase (MAPK)/RAS pathway, and increased levels of monocyte chemoattractant protein-1 (MCP-1) [21], contributing to arterial remodeling and dysplasia. Arterial involvement most commonly affects the renal, cerebral and mesenteric arteries, with RAS representing the most common extra-cranial vascular lesion [10, 14, 21]. Patients with pathogenic *NF1* variants have been found to present with pRVH earlier and with a more severe phenotype [22]. For example, the p.K1444E variant has been associated with increased cardiovascular abnormalities in patients with NF-1, including pRVH [15, 28].

### Histopathologic Findings in NF-1

Histopathologic evaluations of RAS in patients with NF-1 have revealed characteristic features including intimal fibroplasia which may be concentric or eccentric, disruption of the internal elastic lamina, and thinning of the tunica media [22]. Compared to other causes of pRVH, patients with NF-1 may also exhibit non-constricting neural elements within the outer adventitia of affected arteries, as well as extrinsic arterial compression related to plexiform neurofibromas [29]. Intimal hyperplasia and medial thinning are commonly observed in pediatric patients with focal stenotic lesions more broadly [29–31].

These findings contrast with the pathology of adult FMD, which typically presents as multifocal disease with medial fibroplasia, disorganized smooth muscle cell orientation, and accumulations of extracellular matrix collagens [31]. In a comprehensive histologic study, *Coleman et al.* defined intimal fibroplasia as a consistent feature of pediatric RAS [29]. Similar histologic patterns have been observed in other genetic syndromes associated with pRVH, including Alagille syndrome (due to pathogenic variation in *JAG1* or *NOTCH2* genes), Williams syndrome (microdeletion involving the *ELN* gene), and Moyamoya disease [30]. Additionally, intraparenchymal vascular disease appears to be less common in patients with NF-1 than in other forms of pRVH [31].

### Imaging and Diagnosis

pRVH is often first identified after an asymptomatic elevated blood pressure reading [7, 8, 10, 11], though it may also present with symptoms such as headache, chest pain, abdominal pain with or without abdominal bruits, shortness of breath, failure to thrive, seizures, or heart failure [7, 32–34]. The American Academy of Pediatrics recommends routine blood pressure screening in all children beginning at age three years, and earlier for children with genetic syndromes associated with hypertension, including NF-1 [35]. Hypertension occurs in approximately 16–19% of patients with NF-1 [21]. However, vascular lesions can occur even in the absence of sustained hypertension, with some cases only recognized through ambulatory blood pressure monitoring (ABPM) [10, 36], underscoring the value of incorporating ABPM screening in early detection in the NF-1 patient population. It is important to note that in the absence of confirmed hypertension, further work-up, including imaging, is not indicated.

Imaging and diagnostic tests should be ordered in a uniform manner to evaluate for pRVH. In addition to imaging studies, initial laboratory tests should be performed to assess for signs of renal dysfunction (e.g., azotemia, elevated creatinine) or electrolyte derangements such as hyponatremia, hypokalemia, and alkalosis [35]. These electrolyte abnormalities are suggestive of RAS. Unilateral RAS may present as hypertensive hyponatremic syndrome (HHS), which may be accompanied by hypokalemia (termed hypertensive hyponatremic hypokalemic syndrome, HHHS), characterized by hyperactivation of the renin-angiotensin-aldosterone system (RAAS) [37]. Isolated hypokalemia may also be seen in unilateral RAS, occurring in the setting of secondary hyperaldosteronism [38]. Bilateral RAS may manifest with decreased renal function due to hypoperfusion, which can be exacerbated by initiation of angiotensin-converting enzyme inhibitors (ACEi) or angiotensin-receptor blockers (ARBs) [39]. Following initiation of antihypertensive therapy in children with RAS, a metabolic panel including creatinine should be checked within one to two weeks to monitor for evolving kidney injury. In cases of unilateral RAS with prolonged ischemia, compensatory hypertrophy of the contralateral kidney may occur, resulting in glomerular hyperfiltration and the development of proteinuria and glycosuria, biomarkers of subclinical damage to an otherwise normal kidney [39].

Once hypertension has been identified, confirmation of renovascular etiology requires imaging. Imaging modalities to confirm the diagnosis of RVH include duplex ultrasonography, computed tomography angiography (CTA), magnetic resonance angiography (MRA), and catheter based digital subtraction angiography (DSA) [7, 40]. CTA offers excellent spatial resolution and high sensitivity and specificity (90.0% and 89.7%, respectively) [41], but carries risks related to radiation and contrast. MRA avoids radiation but may require sedation and carries a small risk of nephrogenic systemic fibrosis [42]. Duplex ultrasound can be useful for detecting differences in kidney size, identifying main renal artery stenosis and excluding obstructive uropathy when performed at centers with expertise; this diagnostic modality is operator- and patient-dependent and less sensitive for detecting branch or parenchymal disease [1, 43]– [44]. For pRVH, it is better used for surveillance of known and/or previously treated lesions and longitudinally tracking renal growth rather than confirming a diagnosis of renovascular hypertension. Despite advances in noninvasive imaging, DSA remains the gold standard, especially in conditions such as NF-1, where pre-procedural imaging may be inconclusive [45] and arterial involvement may involve occlusive disease such as in the main renal artery or branches, as well as aneurysmal remodeling. Emerging modalities such as photon-counting CT and time-of-flight MRI show promise for improving spatial resolution with lower radiation or without contrast [46]– [47].

The plasma renin activity (PRA) level is often included in the diagnostic workup but is influenced by multiple factors including age, sodium intake, posture, and time of day, making interpretation challenging. PRA may also be suppressed in primary (essential) hypertension, especially in African American patients, and in monogenic forms of hypertension (e.g., Liddle syndrome). Studies have demonstrated normal PRA values in 20–37% of patients with unilateral RAS [48]. In bilateral RAS, children are more likely to have normal renin and aldosterone levels due to volume-dependent hypertension, where initial RAAS activation is followed by volume retention and subsequent renin suppression. Given the low predictive value of PRA, further investigations should be pursued if there is a high index of clinical suspicion for pRVH. Additional evaluations, including echocardiography and fundoscopy, should be performed to assess for end-organ damage after diagnosis [49]. Serial surveillance recommendations vary according to the burden of arterial involvement.

### Medical Management of Hypertension

In children who have a confirmed diagnosis of hypertension and are currently undergoing work-up for pRVH, they should be started on medical management of their hypertension. Medical management should target a blood pressure goal below the 90th percentile for age, sex and height [35]. Medical therapy is the first-line approach for managing confirmed pRVH as well. Angiotensin converting enzyme inhibitors and ARBs may be used with caution, particularly in cases of main renal artery stenosis. However, they are generally avoided in patients with bilateral disease or unilateral disease with a single kidney, due to the risk of acute kidney injury; thus ACEi or ARB treatment should be started only after bilateral RAS or unilateral RAS to a single kidney is excluded [10]. Alternate first-line agents include calcium channel blockers and beta-blockers [49]. Children with resistant hypertension often require combination therapy, which may include additional classes such as peripheral and central alpha-antagonists, direct vasodilators, and diuretics [6, 39, 49]. Diuretics should be considered as adjuncts, as their exclusive use may increase blood pressure [10].

In a series by *McTaggart et al.* three patients with NF-1 and pRVH demonstrated improved blood pressure control with anti-hypertensive therapy alone [6]. While permissive hypertension is sometimes tolerated to optimize renal perfusion, pharmacologic therapy may not be adequate in mitigating end-organ damage or achieving target blood pressures (below the 90th percentile for age, height, and sex in children and below 130/80 mmHg in adolescents and young adults over the age of thirteen) for which endovascular or surgical revascularization may be required (Table 2) [35]. Once patients have a confirmed diagnosis of pRVH and have undergone imaging, the feasibility of endovascular and surgical reconstruction is able to be discussed.

**Table 2:** Clinical series describing revascularization for NF-1 related pRVH

Clinical Series Author and PMID	Years of Study	Clinical Series Location	*N*_NF−1_/*N*_Total_	Intervention	Age of Intervention NF-1	Follow-up Timeline (for all patients)	Outcomes for NF-1 Patients	Series Outcomes
*Alexander et al.* [50] 27634559	1992–2009	University of Toronto Hospital for Sick Children, Canada	5/28	Angioplasty	Subgroups not reported separately	At least 1y. Median 3.8y (1.0–9.0.9y)	3/5 patients had clinically improved blood pressure	10/28 patients had cure, 9/28 patients had improved BP, 9/28 patients had treatment failure
*Bayrak et al. *[51] 19241923	1991–2006	Dicle University, Turkey	3/20	Angioplasty	Subgroups not reported separately	Mean: 55.7 m	1/3 patients had a technically successful result; 2/3 patients had a delayed clinical response; 1/3 had restenosis	Takayasu arteritis: 21/24 patients had a technically successful response and 5/12 had restenosis; Fibromuscular dysplasia: 7/7 had a technically successful result and 1/5 had restenosis
*Booth et al.* [52] 12105246	1982–2000	Guy’s Hospital, England	6/6	Angioplasty	4-15 years	Data not provided	33% success rate on patients with primary stenosis; hypertension improvement in 33%, and 67% on stenoses post-surgery	N/A
*Coleman et al.* [9] 32276020	1991–2017	University of Michigan, USA	31/169	Open surgical repair	Subgroups not reported separately	Mean: 49 m	9/31 had cure, 16/31 had improvement, and 6/31 had failure	65/138 patients without NF–1 had cure, 62/138 had improvement, and 11/138 had failure
*Fossali et al.* [10] 10955932	Published in 2000	Università di Milano, Italy	3/3	Angioplasty	6.3–10.4 years	2y follow-up	Hypertension cure in 3/3 patients	N/A
*Kari et al.* [53] 25527520	1984–2012	Great Ormand Street Hospital, England	19/78	Angioplasty	Subgroups not reported separately	Median: 6y (0.6-16y)	Hypertension cure in 6/19 patients, improvement in 10/19 patients and no improvement in 3/19 patients	14/78 had cure, 31/78 had improvement after one treatment
*Kim et al.* [12] 33340698	2010–2018	Boston Children’s Hospital, USA	6/39	Open surgical repair	Subgroups not reported separately	Median: 2.5y (1.2–5y)	Separate results for NF-1 not provided	31/39 were normotensive at last follow up
*McTaggart et al.* [6] 10975320	1975–1996	Royal Children’s Hospital, Australia	6/10	Open surgical repair	4 months-18 years	Range: 5d-20.4y	3/6 patients experience cure; 1/6 patient improved; 2/6 patient experienced treatment failure and death	2/2 patients with mid-aortic syndrome improved and 2/2 patients with fibromuscular dysplasia cured
*Raborn et al. *[54] 31672102	Published in 2019	University of Alabama Birmingham, USA	2/2	Angioplasty	4–8 years	Range: 6 m-1 y	Hypertension improvement in 2/2 patients	N/A
*Salice et al.* [55] 35983863	2016–2018	Università di Milano, Italy	2/13	Cutting Balloon Angioplasty	9.8–19.8 years	1y follow-up	Hypertension improvement in 2/2 patients	100% achieved technical success; hypertension cured in 7/11 patients; hypertension improved in 4/11 patients
*Srinivasan et al.* [31] 20884235	1997–2009	Children’s Hospital of Philadelphia, USA	7/19	Angioplasty	3.3–17.9 years	Range: 1–83 m	Hypertension cure or improvement in 5/7 patients	Hypertension cure or improvement in 5/11 patients with fibromuscular dysplasia
*Stadermann et al.* [56] 19846390	1979–2008	Great Ormand Street Hospital, England	6/37	Surgical Repair	Subgroups not reported separately	Median: 5.0y (1.4–16y)	Separate results for NF-1 not provided	43% of patients cured of hypertension, 41%improved; 11% of patients had no change in hypertension
*Yousseff et al.* [57] 40164847	2014–2023	Emory University, USA	6/28	Angioplasty	Subgroups not reported separately	Mean: 2.5y (4d – 10.4y)	No significant difference in the number of angioplasty procedures required between patients with NF-1 and FMD	Across the series, 57% achieved hypertension cure and 43% improvement (requiring reintervention in 12 patients)

Studies containing a minimum of 2 or more patients with NF-1 included in report. N_NF−1_ represents the number of patients with NF-1 diagnoses, N_Total_ represents the total number of patients in the series, *d* days, *m* months, *y* years, PMID represents the PubMed identifier

### Percutaneous Transluminal Renal Angioplasty

Endovascular therapy is increasingly utilized in the management of RAS complicated by pRVH in children. Lesions in the mid-distal renal artery or those that are multifocal may benefit most from angioplasty, while ostial or complex lesions, among other presentations, may be better suited to open surgical repair [9]. Some reports suggest poor success of percutaneous transluminal angioplasty (PTA) in patients with NF-1, while others demonstrate outcomes comparable to those in children with non-NF-1 related pRVH, most often with transient or modest benefits [31, 50, 53, 57, 58].

Across studies, clinical cure (defined as restoration of normotension off all anti-hypertensive medications) following PTA in pediatric patients ranges from 18 to 60% [31, 50, 59, 60]. In a clinical series of PTA for RAS by *Srinivasan et al.*, seven patients with NF-1 underwent angioplasty for short (< 10 mm) renal artery lesions, four of which were ostial. Hypertension improved or was cured in five of the seven cases, with success rates similar to those seen in non-NF-1 patients. Follow-up intervals ranged from 1 to 83 months [31]. *Kari et al.* reported on 19 patients with NF-1: six were cured, and ten experienced improvement at most recent follow up (ranged between 0.6 and 16 years). The best outcomes in this series were observed in patients with unifocal stenosis of the main renal artery [53]. A literature review by *Srinivasan et al*. found historical success rates of 54–94% for PTA in NF-1 and fibromuscular dysplasia between 1987 and 2006 [31]. Branch artery disease was associated with higher reintervention rates over time compared to main artery lesions [32]. The impact of lesion length on PTA success remains unclear, with conflicting findings in the literature [55, 59, 60]. One challenge with aggregating clinical cure is that the majority of studies evaluate patients at most recent follow-up, which typically ranges between short-term and long-term outcomes.

Stents should be avoided whenever possible in children. As current stents do not accommodate growth, they may become sites of future stenoses and are subject to additional complications like mechanical failure (ie: stent fracture, kinking) and thrombosis that make the routine use of stents to treat RAS strongly discouraged [9, 44]. Complications of PTA include vasospasm, renal infarction, and perforation [9], the rates of which have not been reported to be higher in patients with NF-1 [52]. Cutting balloon angioplasty incises the intima and media, offering more precise control when dilating stenoses and is useful for lesions resistant to PTA with a bland balloon [44]. In a series by *Salice et al.*, this technique was successful in all 13 patients with pRVH, including two with NF-1, and led to a reduction in anti-hypertensive use [55]. Compared to PTA, cutting balloon angioplasty may be advantageous, especially when intimal hyperplasia limits PTA success [31, 61]. However, it carries increased risks such as dissection, extravasation, and renal artery aneurysm, requiring careful sizing and, if needed, follow-up with balloon angioplasty [44, 58].

### Surgical Intervention

Surgery is indicated for pRVH following failed angioplasty or as a first-line approach to unifocal, long-segment, bifurcation or branch lesions, MAS, or stenoses with associated complex aneurysms [9, 12, 62]. Techniques include renal artery reimplantation, aorto-renal or ileorenal bypass, renal auto-transplantation, and when necessary, nephrectomy. In a series by *Eliason et al.* of patients with remedial operations after PTA, surgery led to hypertension cure in 25% and improvement in 54% of 24 children at follow-up, which averaged 2.1 years [62]. In a series of 169 patients with renovascular hypertension, *Coleman et al.* found that patients with isolated RAS had higher cure rates. They also found that those requiring aortic reconstruction, especially with NF-1, had lower response rates and higher reoperation frequency at mean follow-up of 49 months [9]. In NF-1, optimal outcomes often require a combination of surgical and endovascular interventions [52, 63].

Long-term durability and accommodation for growth are important considerations in surgical planning. Prosthetic grafts do not grow with pediatric patients, making delayed surgery in adolescence preferable when feasible [34]. Emerging techniques such as MAGIC (mesenteric artery growth) and TESLA (tissue expander-stimulated lengthening of arteries) may offer autogenous alternatives [12]. In refractory cases or for those patients with end-stage renal disease, nephrectomy or renal transplantation may be required [9, 64]. Despite variability in approach, both surgical and endovascular interventions have been shown to improve blood pressure control and optimize cardiac remodeling on follow-up imaging [65].

### Potential Future Applications of Targeted Therapies

Targeted therapies such as MEK inhibitors have shown promise in the treatment of non-malignant manifestations of NF-1, particularly plexiform neurofibromas and low-grade gliomas [66]. These therapies act by inhibiting the RAS/MAPK signaling pathway which is dysregulated in NF-1 and contributes to cellular hyperproliferation of the intimal hyperplasia lesion in aorto-renal arterial dysplasia [26]. Although pediatric arterial dysplasia and RVH are not current indications for MEK inhibitor therapy, there is growing interest in their potential utility for vascular complications in NF-1. Preclinical models and case reports suggest a possible role, with arterial remodeling in response to experimental injury shown to be excessive with over-proliferation of vascular smooth muscle cells and interactions of the vascular wall with monoctyes and macrophages [67–73]. However, at current time, clinical evidence in support of the use of MEK inhibitors remains limited.

Sirolimus-coated stents have been highly effective at reducing restenosis following coronary intervention, by inhibiting smooth muscle cell proliferation and thus reducing neointimal growth; however the cellular proliferation in NF-1 is a longer-term process and the targeting of mTOR signaling may not have the same benefit in NF-1 [74]. While mTOR signaling is hyperactivated in NF-1 deficient tumors, this has not been shown in arterial disease, and a clinical trial of sirolimus for non-progressive NF-1 associated plexiform neurofibromas did not show a benefit [75]. Further studies are needed to evaluate the safety, efficacy, and optimal timing of such therapies in children with NF-1-related renovascular disease.

## Conclusion

While there is a growing literature on pRVH and NF-1, much still remains unknown about the natural history, optimal management, and long-term outcomes of pRVH in the context of NF-1. Though rare, untreated pRVH poses significant risks, including chronic kidney disease and other end-organ sequalae. There is an urgent need for research that defines disease progression, refines diagnostic pathways, and identifies effective, individualized treatment strategies. The variability in clinical outcomes and the inherent challenges of conducting randomized trials in rare diseases complicate the development of standardized guidelines. Key priorities include defining the epidemiology and trajectory of NF-1 associated arterial disease, determining the optimal timing and methods for revascularization, and leveraging genetic and molecular insights to guide targeted therapies to address arterial dysplasia. Importantly, insights gained from NF-1 may inform care of other genetic vasculopathies and underscores the value of individualized genotype-specific approaches. Advancing precision medicine in this space is expected to improve outcomes for patients with NF-1 and serve as a model for individualized care across the spectrum of pediatric renal artery disease.

## Key References

- Coleman DM, Wang Y, Yang ML, Hunker KL, Birt I, Bergin IL, Li JZ, Stanley JC, Ganesh SK. Molecular genetic evaluation of pediatric renovascular hypertension due to renal artery stenosis and abdominal aortic coarctation in neurofibromatosis type 1. Human Molecular Genetics. 2022 Feb 1;31(3):334−46. doi: 10.1093/hmg/ddab241.
  - ◦ This study describes the molecular, genetic, and histologic evaluation of pediatric renovascular hypertension in thirteen children with NF-1, reporting new genetic variants in the *NF1* gene that had not previously been described and molecular and histologic features consistent with an aggressive vascular remodeling process.
- Na B, Shah SR, Vasudevan HN. Past, present, and future therapeutic strategies for NF-1-associated tumors. Current Oncology Reports. 2024 Jun;26(6):706−13.
  - ◦ This review summarizes the current innovations in NF-1 treatment with immunotherapy and pharmacotherapy which may have implications for the management of NF-1 associated renovascular hypertension.
- Redhead EC, Paessler A, Arslan Z, Patel P, Minhas K, Forman C, Hollis P, Lava S, Ionescu F, Manuel D, Ray S. Cardiovascular outcomes improve in children with renovascular hypertension following endovascular and surgical interventions. Pediatric Nephrology. 2024 Feb;39(2):521 − 30.Redhead EC, Paessler A, Arslan Z, Patel P, Minhas K, Forman C, Hollis P, Lava S, Ionescu F, Manuel D, Ray S. Cardiovascular outcomes improve in children with renovascular hypertension following endovascular and surgical interventions. Pediatric Nephrology. 2024 Feb;39(2):521−30.
  - ◦ This retrospective review describes outcomes for 152 patients with pediatric renovascular hypertension who underwent an endovascular and/or surgical intervention. It demonstrated significant improvements between pre- and post- echocardiographic parameters following intervention.
- Salice P, Mircoli L, Butera G, Burdick L, Borzani I, Mastrangelo A, Ardissino G, Beretta C, Ferraresso M, Ughi L, Montini G. Percutaneous cutting balloon angioplasty for the treatment of renovascular hypertension in children and adolescents. *Journal of Hypertension*. 2022 Oct 1;40(10):1902–8.
  - ◦ This article describes use of the percutaneous cutting balloon in pediatric renovascular hypertension and included two patients with NF-1. The cutting balloon was technically successful in all patients and resulted in significant reductions in mean blood pressure.

## Data Availability

No datasets were generated or analysed during the current study.
